# Radical-Induced Low-Field ^1^H Relaxation in Solid Pyruvic Acid Doped with Trityl-OX063

**DOI:** 10.1021/acs.jpclett.2c02357

**Published:** 2022-10-31

**Authors:** Michael Jurkutat, Hana Kouřilová, David Peat, Karel Kouřil, Alixander S. Khan, Anthony J. Horsewill, James F. MacDonald, John Owers-Bradley, Benno Meier

**Affiliations:** † Institute of Biological Interfaces 4, Karlsruhe Institute of Technology, Eggenstein-Leopoldshafen76344, Germany; ‡ School of Physics and Astronomy, University of Nottingham, NottinghamNG7 2RD, U.K.; ¶ Institute of Physical Chemistry, Karlsruhe Institute of Technology, Karlsruhe76131, Germany

## Abstract

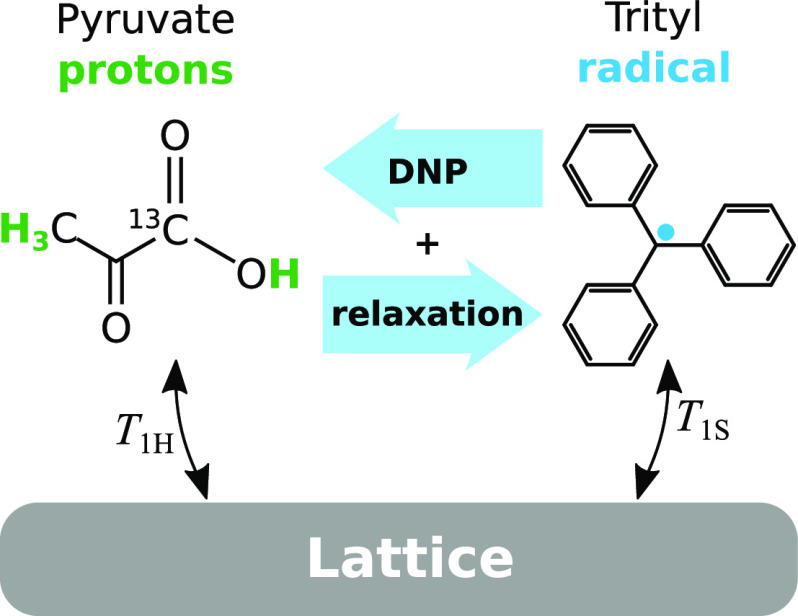

In dynamic nuclear polarization (DNP), radicals such as trityl provide a source for high nuclear spin polarization. Conversely, during the low-field transfer of hyperpolarized solids, the radicals’ dipolar or Non-Zeeman reservoir may act as a powerful nuclear polarization sink. Here, we report the low-temperature proton spin relaxation in pyruvic acid doped with trityl, for fields from 5 mT to 2 T. We estimate the heat capacity of the radical Non-Zeeman reservoir experimentally and show that a recent formalism by Wenckebach yields a parameter-free, yet quantitative model for the entire field range.

Nuclear magnetic resonance (NMR) and magnetic resonance imaging (MRI) are powerful noninvasive techniques used to study the structure and dynamics of matter. At room temperature, however, only 1 in 100,000 ^1^H spins contributes to the NMR signal in a 3 T MRI scanner. The low sensitivity of magnetic resonance can be alleviated by dissolution-dynamic nuclear polarization (D-DNP),^1−4^ a technique first reported by Ardenkjær-Larsen and co-workers.^5^ In D-DNP, nuclear spins are polarized to a high degree using free radicals and microwave irradiation, typically at a temperature of 1 K. The sample is then dissolved with hot solvent, and the solution is transferred to a secondary magnet for detection. In MRI, D-DNP has enabled the *in vivo* observation of human metabolism.^6−9^

We have recently demonstrated a different scheme, named bullet-DNP, where the hyperpolarized solid is transferred to the secondary magnet within 100 ms and dissolved only near the point of use, giving rise to ^13^C liquid-state polarization in excess of 30%.^10,11^ A magnetic tunnel along the entire transfer path is used to establish a minimum magnetic field of approximately 70 mT. Bullet-DNP is fast and scalable toward small solvent volumes as they are used in NMR spectroscopy.^12^ It may also be useful for the transfer of hyperpolarized substances where the *T*_1_ in liquid-state is short, as is the case for high-γ nuclei and larger molecules.

While the radicals are the source of polarization in DNP, their presence also causes nuclear relaxation, in particular at low field. A previous study by Niedbalski et al.^13^ on low temperature relaxation between 0.9 and 9 T in solid pyruvic acid doped with trityl-OX063 found a cubic field-dependence of the relaxation time constant *T*_1,C_ for ^13^C. Such a trend cannot persist to lower fields, as relaxation would become prohibitively fast for the transfer in bullet-DNP.^10^ Low-field relaxation due to residual radicals is also highly relevant for a range of recent DNP applications^14,15^ that involve the transfer of hyperpolarized solids.

Here, we show that the low-field *T*_1,H_ of ^1^H scales linearly with the applied magnetic field. The data are analyzed using a thermodynamic spin temperature model,^16−18^ wherein commuting terms in the Hamiltonian are described as reservoirs with associated heat capacities and temperatures. Weak couplings between the reservoirs give rise to heat exchange. The nuclear spin Zeeman reservoir is coupled to the electron dipolar or Non-Zeeman (NZ) reservoir via energy-conserving triple-spin-flips (TSFs). These TSFs comprise a nuclear spin-flip and a simultaneous electron–electron flip-flop. The associated *heat exchange rate* τ^–1^ due to TSFs is, in shorthand, referred to as triple-spin-flip rate.^19^ We calculate the TSF rate from first-principles,^20,21^ using only a Monte Carlo based estimate of the spectrum of electron spin–spin interactions,^22^ and the inhomogeneous EPR line width from experimental electron paramagnetic resonance (EPR) data.^23^ Our experimental nuclear relaxation data and the TSF rate are combined to derive a correction of the Monte Carlo based spin–spin spectrum, which leads to quantitative agreement between the model and the measured relaxation rates for protons over 2 orders of magnitude in the magnetic field.

The experiments were performed using a fast-field-cycling setup^24^ that allows for fast field switches and enables the measurement of relaxation phenomena down to 0 T. The samples investigated are nondegassed neat 1-^13^C pyruvic acid (neat PA, □) as well as 1-^13^C pyruvic acid doped with 15 mM OX063 (doped PA, ■). Measurements were done at fields between 5 mT and 2 T and at temperatures between 4.2 and 40 K. Experimental details can be found in the Supporting Information (SI).

The field-dependence of the proton relaxation time for doped PA, *T*_1,H_^■^ is shown in Figure 1(a), for temperatures between 4.2 and 40 K. At low fields, we find a linear increase of *T*_1,H_^■^ with field. As the temperature is increased from 4.2 to 20 K, *T*_1,H_^■^ decreases slightly. From 20 to 40 K, *T*_1,H_^■^ decreases significantly, which may be attributed to methyl-group-induced relaxation.^25−27^

**Figure 1:**
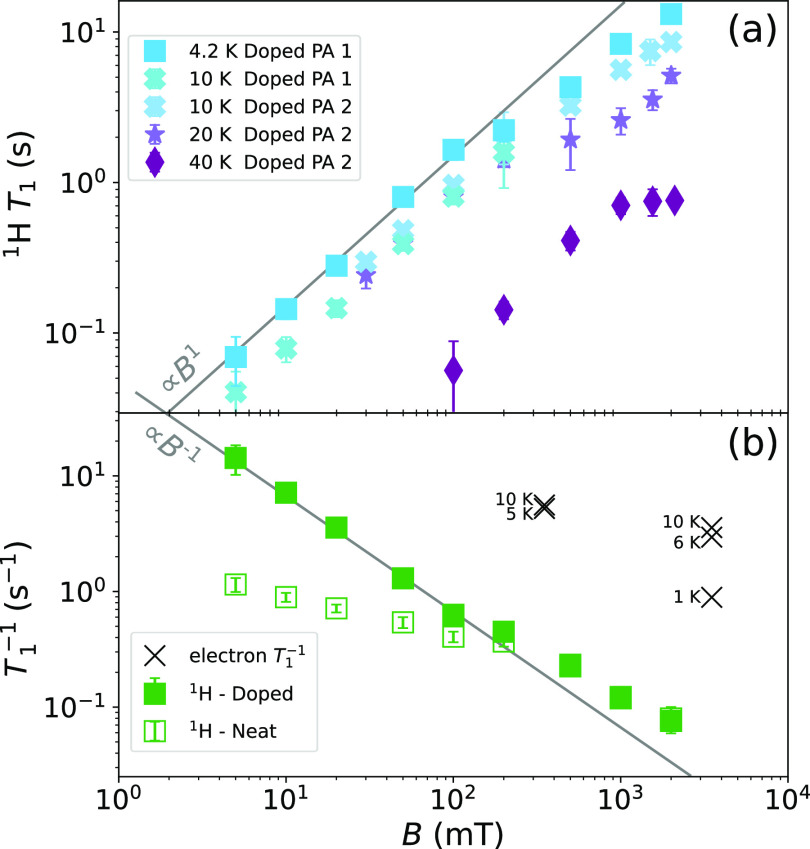
Field-dependent proton relaxation in 1-^13^C PA doped with 15 mM trityl (OX063) from 5 to 2000 mT. (a) Relaxation time constants of two equivalent samples measured at different temperatures. The gray line is a guide to the eye according to *T*_1,H_ = *cB*_0_^1^ with *c* = 15 s/T. The 4.2 K data from panel (a) are plotted again as rates in panel (b) (solid symbols), where they are compared to the relaxation rates measured on neat PA at 4.2 K (open symbols). Also shown are literature data^23^ on the electron *T*_1,S_ for doped PA at temperatures near 4 K.

We compare relaxation rates 1/*T*_1,H_, recorded at 4.2 K, for neat and doped PA in Figure 1(b). At low field, trityl enhances relaxation by more than an order of magnitude. The effect decreases with increasing field and the rates for doped and neat PA converge–both materials exhibit similar relaxation time constants *T*_1,H_^■^ ≈ *T*_1,H_^□^ above 500 mT going up to ∼10 s at 2 T and 4.2 K. This value is consistent with an earlier study^28^ and linked to the presence of oxygen in nondegassed PA.

Also shown in Figure 1(b) are electron spin–lattice relaxation rates reported by Lumata et al.^23^ for doped PA at temperatures near 4.2 K. These indicate an approximately field-independent *T*_1,S_^–1^ ≈ 5 s^–1^. A significant field-dependence of the electron *T*_1,S_ is not expected, since trityl relaxes predominantly via oxygen.^29^

We analyze the trityl-enhanced relaxation in the framework of a thermodynamic spin-temperature model, where relaxation is modeled as a flow of heat between different spin reservoirs and the lattice,^30^ as indicated in Figure 2. Following a perturbation, each reservoir quickly achieves an internal equilibrium, which is described by a spin temperature. The heat capacities of each reservoir are obtained as derivatives of their energy with respect to inverse temperature *β*_*i*_ = ℏ/*k*_B_*T*_*i*_.^30^ Note that *β*_*i*_ is a measure of a spin reservoir’s polarization , with the Larmor frequency ω_*i*_. The relevant reservoirs for the system discussed here are the nuclear Zeeman reservoirs and the electron Non-Zeeman (NZ) reservoir. These three couple to each other and the lattice as sketched in Figure 2. The electron Zeeman reservoir may couple to these reservoirs, if the difference in Zeeman energies of two electron spins approaches the nuclear Zeeman energy. Owing to the small *g*-anisotropy of trityl, this effect may be ignored here. Likewise, the nuclear Non-Zeeman reservoirs are ignored due to their small heat capacity.

**Figure 2:**
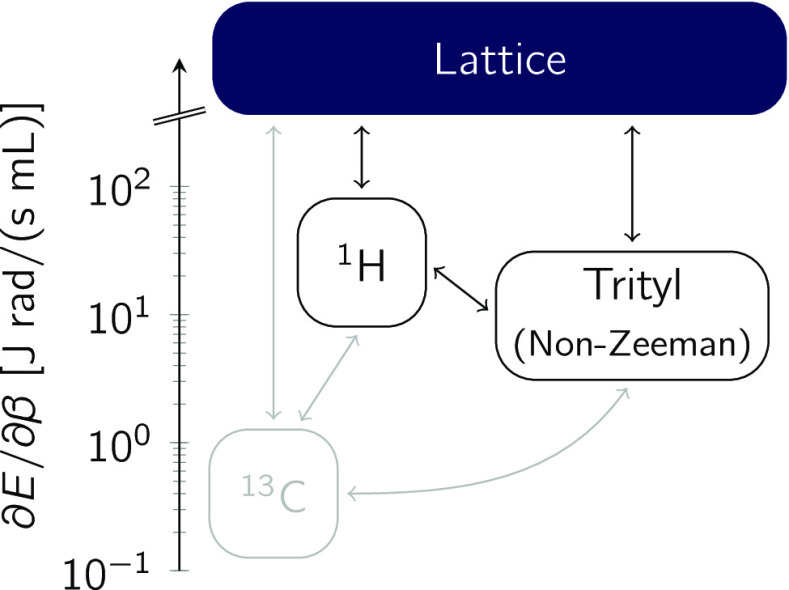
Heat capacities of the ^1^H (and the ^13^C) nuclear Zeeman reservoir for pyruvic acid at *B*_0_ = 20 mT and the trityl Non-Zeeman reservoir. All reservoirs exhibit field-dependent couplings to each other and the lattice.

For the nuclear Zeeman reservoirs with energies *E*_*i*_, with *i* ∈{^1^H, ^13^C}, and the NZ reservoir with *E*_NZ_, the heat capacities are, respectively,
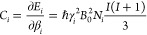
1
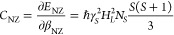
2where *B*_0_ is the applied magnetic field, γ_*i*_ is the gyromagnetic ratio, and *N*_*i*_ is the concentration of the respective nuclear spins. The local field *H*_*L*_ due to spin–spin interactions is given by *γ*_*S*_^2^*H*_*L*_^2^ = (5/3)*M*_2_, where *M*_2_ is the second moment of the dipolar EPR line, *N*_*S*_ is the radical concentration, and *I* = *S* = 1/2 is the spin of the involved species.^30^ The Zeeman heat capacities scale quadratically with the applied field, whereas the NZ heat capacity is field-independent. For 1-^13^C PA, the heat capacity of the carbon Zeeman reservoir, at any given field, is  times smaller than the proton heat capacity, and so its influence on the other reservoirs is negligible. Vice versa, the carbon Zeeman reservoir may be influenced strongly by both the proton Zeeman and the NZ reservoir. A preliminary analysis of the carbon relaxation is reported elsewhere.^31^

The proton reservoir with inverse temperature β_H_ couples to the NZ reservoir (with the rate ) and the lattice (), while vice versa, the NZ reservoir with inverse temperature β_NZ_ couples to the proton reservoir (with the rate τ_NZ–H_^–1^) and the lattice (with *T*_1,S_^–1^). The heat exchange of the proton Zeeman and electron NZ reservoirs with each other and the lattice, depicted in Figure 2, is described by a pair of linear differential equations, which may be written in matrix form as
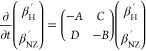
3

Here, *β*_*i*_^′^ = *β*_*i*_ – *β*_L_, with *i* ∈ [H, NZ], is the difference of the respective reservoir’s inverse temperature and the inverse lattice temperature, *β*_L_. The relaxation matrix entries are *A* =  + *C*_NZ_/*C*_H_ · *τ*_NZ–H_^–1^, *B* = *τ*_NZ–H_^–1^ + *T*_1,S_^–1^, *C* = *τ*_NZ–H_^–1^*C*_NZ_/*C*_H_, and *D* = *τ*_NZ–H_^–1^.

*T*_1,H_^□^ in (3) has been measured directly in neat PA, and the proton heat capacity can be calculated using (1). The electronic relaxation rate 1/*T*_1,S_ ≈ 5 s^–1^ of doped PA is known from earlier work by Lumata et al.^23^

To describe the proton relaxation, we need to determine the TSF rate *τ*_NZ–H_^–1^ and the heat capacity of the NZ reservoir. In the high-temperature approximation, the change in NZ energy *E*_NZ_ due to exchange with a nuclear reservoir via TSFs is^21^
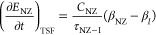
4

Note that TSFs can also exchange electron Zeeman energy with nuclear reservoirs, a process known as the *cross effect*. For the narrow-band OX063 radical, however, the inhomogeneous EPR line width is much smaller than the proton Larmor frequency such that the cross effect here is negligible, and we consider only *thermal mixing* with the NZ reservoir.

An expression for the left-hand side of eq 4 has recently been given by Wenckebach.^32,33^ As detailed in the SI, his expression may be rewritten using the high-temperature approximation, leading to the following expression for the TSF rate
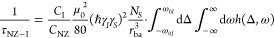
5with the integrand
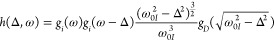
6

The TSF rate thus depends on the radical concentration *N*_*S*_, the distance between the electron and the nearest nuclear spins outside the diffusion barrier *r*_ba_, the spectrum of electron spin–spin interactions *g*_*D*_ (ω), and the inhomogeneous EPR line shape *g*_*i*_ (ω).

The number density is known from the trityl concentration, and we set *r*_ba_ to 0.7 nm.^33^ Monte Carlo simulations^22^ yield a spectrum of electron spin–spin interactions *g*_*D*_ (ω) that can be described by the normalized product of a Gaussian with a Lorentzian, with line widths for our system of Δ_G_/2π = 8.48 MHz and Δ_L_/2π = 1.18 MHz. The second moment *M*_2_ of this spectrum corresponds to a dipolar width of only  = 2.7 MHz, with a corresponding NZ heat capacity of 0.12 J rad/(s mL). For the inhomogeneous EPR line, we assume a Gaussian with line widths interpolated from EPR data reported by Lumata et al.^23^ Further details are given in the SI. The resulting EPR spectrum (*g*_*i*_ * *g*_*D*_) (ω) at 0.35 T is sketched in Figure 3(a).

**Figure 3:**
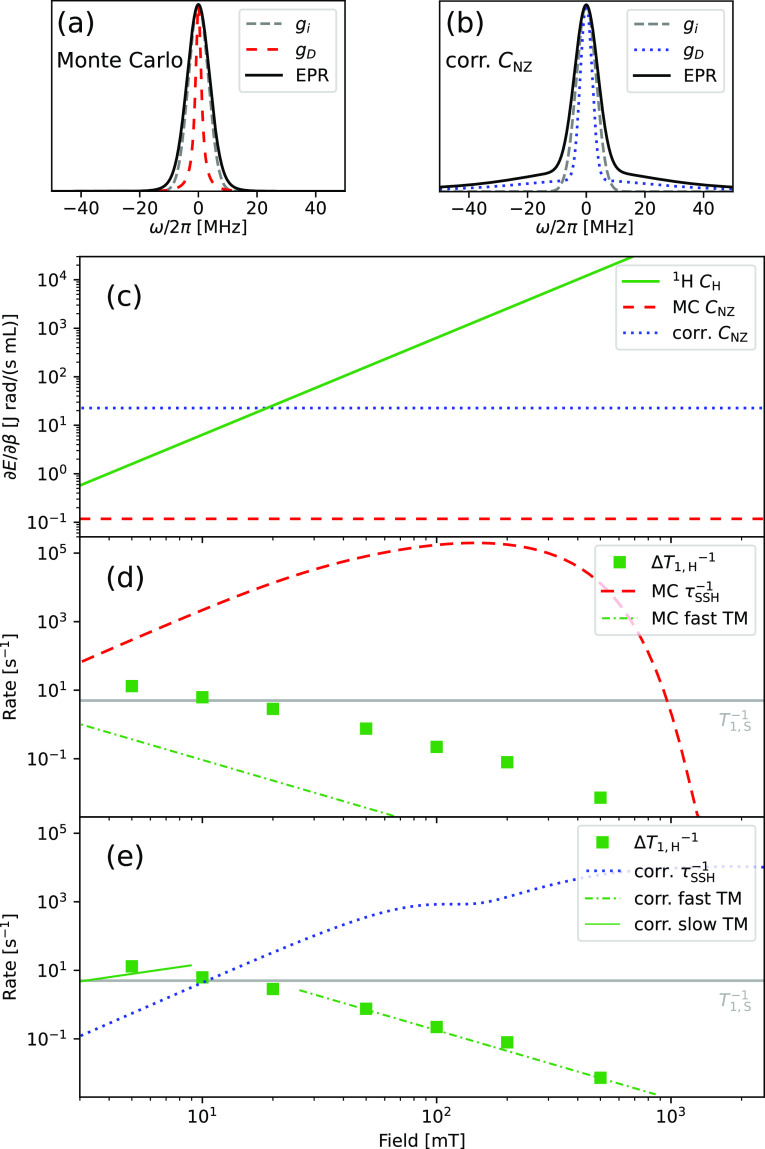
(a) Sketch of the EPR X-band (*B*_0_ = 0.35 T) spectrum (*g*_*i*_ * *g*_*D*_) (ω) with *g*_*D*_ (ω) based on Monte Carlo simulations,^22^ compared to (b) the model with enhanced spin–spin interaction. (c) The ^1^H Zeeman heat capacity *C*_H_(*B*_0_) grows quadratically with field. The NZ reservoir’s heat capacity *C*_NZ_ is constant but depends on *g*_*D*_ (ω). (d) The proton TSF rate (dashed red line) as calulated with the Monte Carlo spin–spin spectrum from panel (a) predicts fast thermal mixing (dash-dot green line according to eq 7) but underestimates the observed increase in proton relaxation rate *ΔT*_1,H_^–1^ by more than 1 order of magnitude. (e) The corrected model from panel (b) yields a TSF rate (dotted blue line) that predicts fast thermal mixing (dash-dot green line) for 30 mT < *B*_0_ < 1 T and slow thermal mixing (solid green line, eq 8) for *B*_0_ ≪ 10 mT, both in quantitative agreement with the experimental data.

The TSF rates are calculated numerically as detailed in the SI. The resulting field-dependent TSF rate for hydrogen *τ*_NZ–H_^–1^(*B*_0_), calculated with the aforementioned Monte Carlo-based parameters, is shown in Figure 3(d), along with the increase in hydrogen relaxation rate due to trityl, Δ*T*_1,H_^–1^ =  – . The TSF rate *τ*_NZ–H_^–1^ exceeds the electron spin–lattice relaxation rate *T*_1,S_^–1^ for fields up to 1 T. Furthermore, as we show in Figure 3(c), the hydrogen heat capacity exceeds that of the NZ reservoir throughout our experimental range, *C*_H_ ≫ *C*_NZ_ for the Monte Carlo model.

Under these conditions, known as *fast thermal mixing*, the NZ reservoir equilibrates rapidly with the proton reservoir, and we have *β*_NZ_(*t*) ∼ *β*_H_(*t*) .^19^ One can deduce from (eq 3) that the increase in proton relaxation rate is then
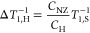
7

Since we assume a field-independent *T*_1,S_^–1^ ≈ 5 s^–1^ and *C*_H_ scales quadratically with *B*_0_, eq 7 predicts an inversely quadratic field dependence of *ΔT*_1,H_^–1^. This dependence is shown as the dash-dot green line in Figure 3(d), where we assume *C*_NZ_ = 0.12 J rad/(s mL) as predicted by the Monte Carlo simulations.^22^

One can see from eq 7 that *ΔT*_1,*H*_^–1^ is limited by the field-independent heat capacity of the NZ reservoir. Experimentally, the additional relaxation due to trityl follows the predicted quadratic field dependence above 20 mT, but the observed rates are approximately an order of magnitude larger than the prediction. Conversely, a good match between (eq 7) and the experimental data is obtained when we assume an NZ capacity of 23 J rad/(s mL). This corresponds to a larger second moment of the electron spin–spin spectrum of  = 38 MHz.

As detailed in the SI, the hyperfine interaction of the trityl electron spin with ^13^C carbon occurring at natural abundance cannot account for the observed second moment.^34^ Instead, we attribute the larger second moment to the reported propensity of trityl radicals to cluster. At elevated radical concentrations, this leads to homogeneous broadening of the EPR spectrum in excess of 100 MHz,^35,36^ well beyond what we report in this study. Note that the precise spatial distribution of radicals and the corresponding EPR spin–spin spectrum cannot be determined from our NMR data, nor are they critical to our analysis. As detailed in the SI, the observed second moment is readily generated by different spin–spin spectra yielding similar TSF rates in our field range, and we choose one close to an experimental EPR spectrum,^23^ as depicted in Figure 3(b).

The TSF rate for protons, recalculated for the spin–spin spectrum *g*_*D*_ with larger heat capacity, is compared to the experimental data in Figure 3(e). As can be seen, the conditions for fast thermal mixing (*C*_H_ ≫ *C*_NZ_, *τ*_NZ–H_^–1^ ≫ *T*_1,S_^–1^) are fulfilled above 20 mT, exactly where the inversely quadratic field dependence is observed.

Now, at the lowest fields, the corrected NZ heat capacity exceeds that of the proton reservoir, cf. Figure 3(c), and the corrected TSF rate is slower than the electron spin–lattice relaxation, cf. Figure 3(e). Then, the NZ reservoir’s temperature remains at lattice temperature (*β*_NZ_^′^ = 0) which is the limiting case of so-called *slow thermal mixing*. In this case, (eq 3) yields
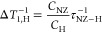
8The expected *ΔT*_1,H_^–1^ according to (eq 8) in that lower field range is indicated by the solid green line in Figure 3(e). While sufficient low field data points are lacking to confirm the expected linear dependence (*C*_H_^–1^ · *τ*_NZ–H_^–1^ ∝ *B*_0_^–2^ · *B*_0_^3^ = *B*_0_^1^), we note the quantitative agreement at 5 mT.

With the NZ heat capacity determined and the TSF rate calculated, we can now obtain a quantitative description over the entire field range by solving (eq 3). The solution may be written as *β⃗* ′(*t*) = ∑_*i*=1_^2^*a*_*i*_ v⃗_*i*_ exp(λ_*i*_*t*) with the eigenvalues λ_*i*_ and eigenvectors *v⃗*_*i*_ of the relaxation matrix. The coefficients *a*_*i*_ are given by the initial conditions.

In our experiments, the NZ reservoir is initially at the lattice temperature, i.e., *β*_NZ_^′^(*t* = 0) = 0, while the inverse proton temperature is finite. As detailed in the SI, the evolution of the two reservoirs can then be written as
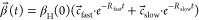
9where the coefficients are
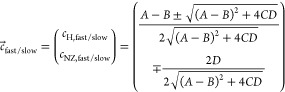
10and the two relaxation rates *R*_fast/slow_ = −*λ*_1,2_ are given by the eigenvalues of the relaxation matrix in (eq 3):
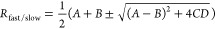
11

Note that a bimodal relaxation is observed whenever two reservoirs are coupled to the lattice and to each other. The fast mode balances the inverse temperatures of the coupled reservoirs, whereas the slow mode describes the equilibration of both reservoirs with the lattice.

The field-dependent proton coefficients and the relaxation rates of the two modes are shown in Figure 4(a) and (b), respectively. In Figure 4(b), we also show the experimentally observed ^1^H relaxation rates for neat and doped PA. Since the experimental data do not warrant an extraction of two coefficients and two decay rates, we compare the experimental data with the effective relaxation rate  evaluated at *t* = *T*_1,H_^■^(*B*_0_), details are given in the SI. As can be seen in Figure 4, the model describes the observed proton relaxation nearly quantitatively.

**Figure 4:**
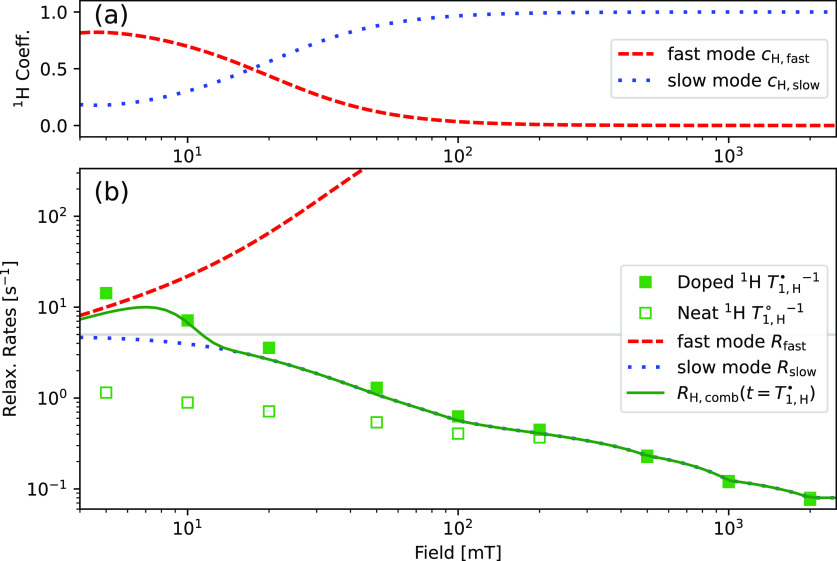
Field-dependent proton relaxation described by bimodal relaxation for two coupled reservoirs, protons and electron NZ. (a) Proton coefficients for the fast and slow mode relaxation. (b) Corresponding fast and slow mode relaxation rates compared to experimental data. Also shown is the coefficient-weighted average relaxation rate *R*_H,comb_ predicted for protons evaluated at *T*_1,H_^■^.

In summary, we have shown quantitative agreement between Wenckebach’s model for the triple-spin-flip rate, and the observed dynamics in the model system pyruvic acid doped with OX063. Thermal mixing dominates in this system, and we find that the NZ reservoir’s heat capacity exceeds that of homogeneously distributed radicals. This effect is attributed to radical aggregation and an associated broadening of the electron spin–spin interaction spectrum. While the details of the spin–spin spectrum are not relevant for the analysis in our field range, they do determine the cutoff field at which the TSF rate vanishes. This finding may support the attribution of ^1^H DNP at a field of 7 T to thermal mixing in DNP substrates with substantial trityl concentrations.^35^

Wenckebach’s theory describes not only thermal mixing but also the cross-effect, and it does not make any assumptions about the used radical. To the contrary, it accounts for all DNP mechanisms that involve triple-spin-flips, which drive the vast majority of D-DNP experiments today. The success of our analysis therefore encourages its extension to other radical/analyte systems, and to DNP experiments at higher fields. A quantitative understanding of the DNP processes will further inform the design of host materials for optimized polarization.

## Supporting Information Available

Details of sample preparation and loading, fast field cycling apparatus and experiments, spin–lattice relaxation data, derivation of triple-spin-flip rate, two-reservoir relaxation, modeled EPR spectra and TSF rates, and python code for TSF rate calculation (PDF)
Transparent Peer Review report available (PDF)
