# Supplementary material for: Radical-Induced Low-Field 1H Relaxation in Solid Pyruvic Acid Doped with Trityl-OX063
Source: J Phys Chem Lett. 2022 Oct 31;13(44):10370–6. doi: 10.1021/acs.jpclett.2c02357 (PMC9661535; doi:10.1021/acs.jpclett.2c02357)
Supplement: Supplementary file 1 — jz2c02357_si_001.pdf [file jz2c02357_si_001.pdf]

Radical-Induced Low-Field ^{1}H Relaxation in Solid Pyruvic Acid Doped with Trityl-OX063: Supplementary Information

Michael Jurkutat,[1,*] Hana Kouřilová,[1] David Peat,[2] Karel Kouřil,[1] Alixander S. Khan,[2]
Anthony J. Horsewill,[2] James F. MacDonald,[2] John Owers-Bradley,[2] and Benno Meier[1,3,†]

[1] *Institute of Biological Interfaces 4, Karlsruhe Institute of Technology, Eggenstein-Leopoldshafen, 76344, Germany*
[2] *School of Physics and Astronomy, University of Nottingham, Nottingham, NG7 2RD, UK*
[3] *Institute of Physical Chemistry, Karlsruhe Institute of Technology, Karlsruhe, 76131, Germany*

(Dated: October 4, 2022)

CONTENTS

I. Sample Preparation 2

II. Sample Loading 2

III. Fast field cycling (FFC) apparatus 2

IV. FFC Experiments 3

V. Spin-lattice Relaxation Data 4

VI. Derivation of Triple-spin-flip Rates 5

VII. Two-Reservoir Relaxation 6
1. Slow relaxation in the high-field limit 7
2. Fast relaxation in the low-field limit 8
3. Interpretation of the two-mode relaxation 8

VIII. OX063 EPR Spectrum and TSF Rates 8

IX. Python code for numerical TSF calculations 10

References 13

I. SAMPLE PREPARATION

Neat 1-^{13}C-pyruvic acid was purchased from CortecNet, FR, and OX063 trityl radical was purchased from Oxford Instruments, UK. Experiments on neat pyruvic acid were carried out on a single sample, referred to as Neat PA, during a single session. Two experimental sessions were required to record data on 15 mM OX063 in 1-^{13}C pyruvic acid. A fresh sample was prepared for each session, and we refer to these samples as Doped PA 1 and Doped PA 2 in this manuscript. All samples were used without degassing.

II. SAMPLE LOADING

For each experiment the corresponding solution was pipetted into either a glass tube and sealed with PTFE tape, or into a PTFE sample cup and closed using a lid with a small hole in its rotation symmetry axis to allow for the pressure equilibration. The sealed tube or the sample cup was inserted into the NMR coil. The samples were flash-frozen by immersion of the NMR probe into the cold variable temperature insert of the magnet.

III. FAST FIELD CYCLING (FFC) APPARATUS

Experiments were carried out using a fast field cycling (FFC) apparatus (Cryogenic Ltd) at the University of Nottingham, UK. The system comprises a low-inductance superconducting magnet and a fast-ramping power supply. The magnet houses a sample flow cryostat and a variable temperature insert (VTI). Stable temperatures in the range of 3 to 300 K and magnetic fields from 0 to 2.5 T are achieved. The magnetic field changes were performed at a rate of 4 T/s, and the absolute error of the magnetic field strength is 2 mT. A Tecmag Apollo NMR spectrometer (Tecmag, TX) with a home-written visual basic extension enables control of the magnetic field strength from within the NMR pulse sequence. Here we used a home-built NMR probe with a solenoid tuned to a frequency of 22.83 MHz. This frequency corresponds to a resonant magnetic field of 0.536 T for protons. A more detailed description of the system has been given by Horsewill et al.[1,2].

IV. FFC EXPERIMENTS

The two pulse sequences used to measure proton relaxation are shown in Fig. S1.

For fields below 0.2 T the polarization from a simple saturation recovery experiment would be too small. Thus, a *polarization decay* sequence that comprises an additional polarization stage (stage II) is used. In this sequence the proton spins are first saturated to ensure reproducibility, and then polarized for 70 s at a magnetic field of 2 T. In stage III the magnetization relaxes to the new thermal equilibrium at the lower magnetic field strength. In stage IV the field is again ramped up to the resonant field and the signal is read out.

For field strengths of 0.2 T and higher a *saturation recovery* sequence is used. The proton magnetization is saturated with a train of typically 1000 2 μs $\pi/2$ pulses (stage I). Stage II in Fig. S1 is skipped. The polarization is allowed to recover for a variable time at a set field (stage III), and the magnetic field is ramped up to the detection field where the signal is read out (stage IV). The relaxation time T_1 is obtained by applying an exponential fit to the integrated spectral intensities.

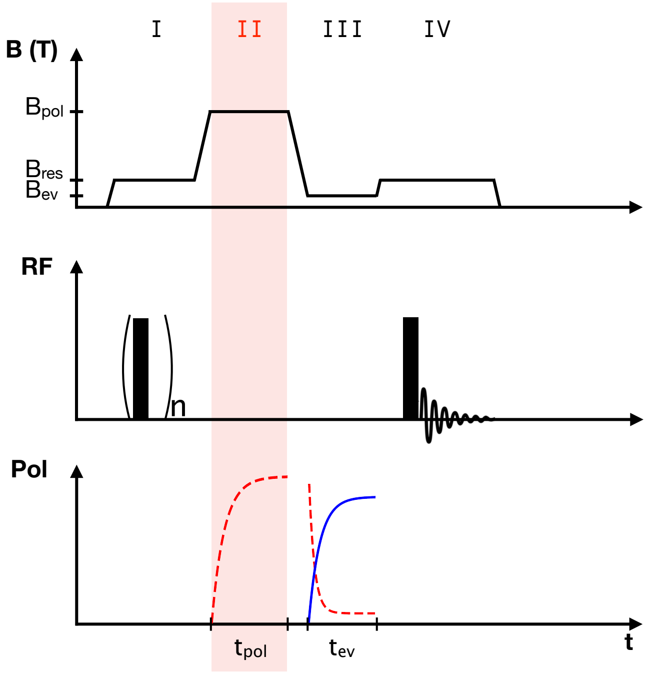

FIG. S1: Pulse sequences used for T_1 measurements. For fields above 0.2 T the magnetization is saturated in stage **I**. Then in stage **II** the nuclei polarize at 2 T (red dashed buildup curve). In stage **III** the field is changed to a set value and the magnetization relaxes (red dashed decay curve). In stage **IV** the NMR signal is read out. For magnetic fields of 0.2 T and higher only stages **I**, **III** and **IV** are used, i.e. stage **II** (highlighted) is dropped. For these fields the magnetization is saturated in stage **I** and recovers (blue buildup curve) in stage **III** at a set magnetic field value. In stage **IV** the NMR signal is read out. B_{pol}, B_{res}, B_{ev}, t_{pol}, t_{ev} and n represent polarization magnetic field, resonance magnetic field, evolution magnetic field, polarization time, evolution time and number of saturation pulses, respectively.

V. SPIN-LATTICE RELAXATION DATA

The experimental data for [1]H T_1 measurements are shown in Figs. S2 and S3 together with exponential fits.

We note that we observe ramp effects on the signal intensities. These are particularly pronounced at low evolution fields where the initial intensities ($t_{\mathrm{evo}} = 0$) appear diminished for [1]H. For long evolution times ($t_{\mathrm{evo}} \gg T_1$) we find signals enhanced. Numerical simulations of the interaction of the nuclear reservoir with the electron non-Zeeman during the ramp, show that the effects are qualitatively reproduced by the energy exchange via the mechanism described in this manuscript, i.e. coupling with a non-nuclear reservoir whose heat capacity is not field-dependent. However, quantitatively the calculated ramp effects account for only about one third of the observed discrepancies. Since we observe similar ramp effects in the neat sample and also for fields above 1 T, we assume that oxygen in the non-degassed neat and doped samples is the origin.

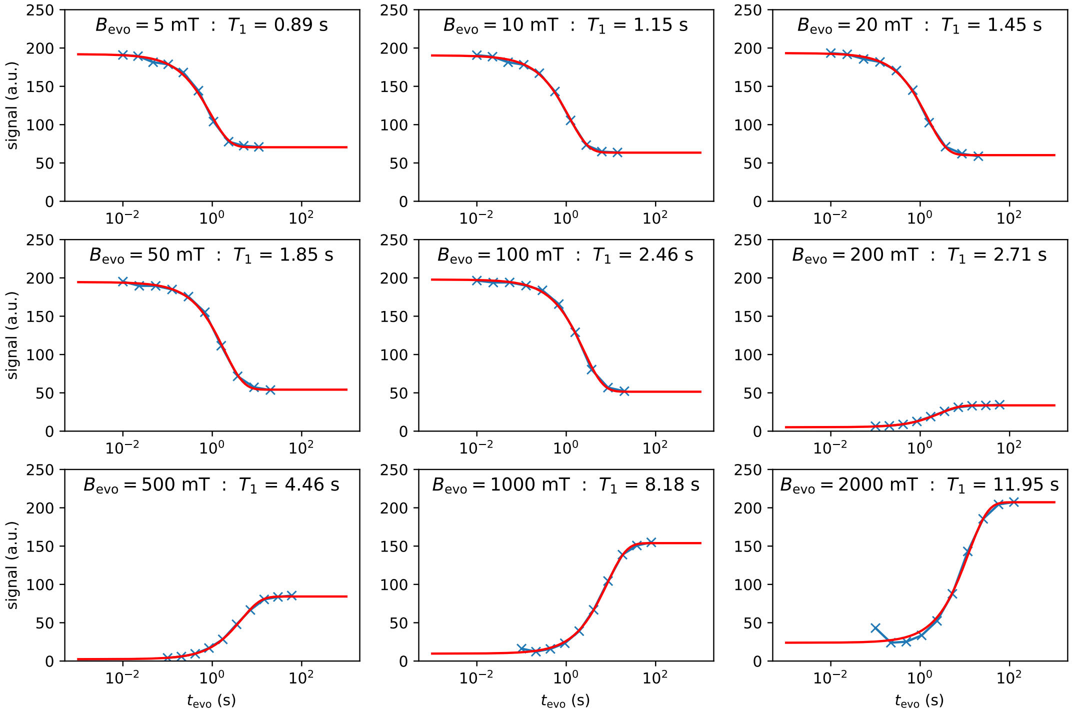

FIG. S2: [1]H T_1 measurements on neat PA at 4 K in different fields using polarization decay up to 100 mT and saturation recovery above.

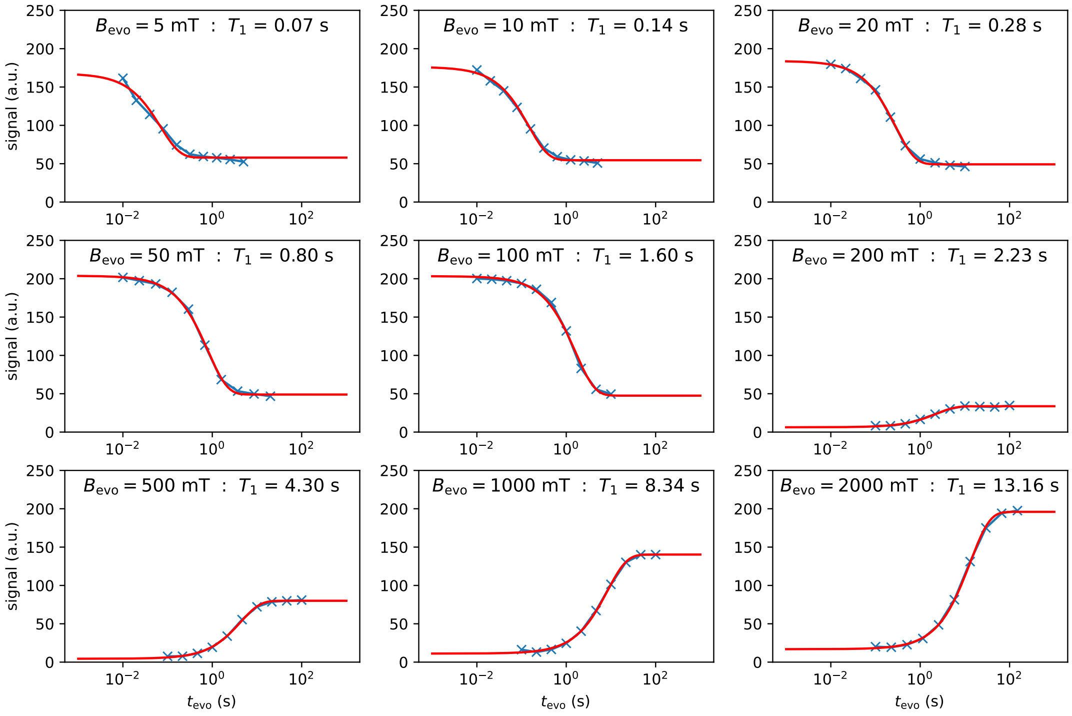

FIG. S3: ^{1}H T_1 measurements on doped PA at 4 K in different fields using polarization decay up to 100 mT and saturation recovery above.

VI. DERIVATION OF TRIPLE-SPIN-FLIP RATES

Wenckebach[3] derives expressions for the energy exchange between nuclei and radicals electrons via triple-spin-flips for both the cross effect (CE) and for thermal mixing (TM). In the CE, nuclear Zeeman energy energy is exchanged with electron Zeeman energy, e.g. due to differences in the g-anisotropy of the two involved electron spins. In TM, nuclear Zeeman energy is exchanged with electron Non-Zeeman energy due to dipolar interactions.[4–6]

We are interested in the latter and start with equation (38) from Ref.[3] for the rate of TM

$$W^{\mathrm{TM}} = \frac{\pi}{2} A^2 \int_{-\omega_{0I}}^{\omega_{0I}} \mathrm{d}\Delta \frac{\left(\omega_{0I}^2 - \Delta^2\right)^{3/2}}{{\omega_{0I}}^3} g_D \left(\sqrt{\omega_{0I}^2 - \Delta^2} \right) , \tag{1}$$

where ω_{0I} is the nuclear Larmor frequency and g_D is the spectrum of electronic spin-spin interactions. The expression for A^2 according to Wenckebach[7] equation (20) is:

$$\begin{aligned} A^2 &= \frac{3}{10} A_0^2 N_I 4\pi \int_{r_{\mathrm{ba}}}^{\infty} \frac{\mathrm{d}r}{r^4} \\ &= \frac{1}{10} A_0^2 N_I 4\pi \frac{1}{r_{\mathrm{ba}}^3}, \end{aligned} \tag{2}$$

where we have carried out the integral over dr. Here r_{ba} refers to the diffusion barrier, N_I is the density of the nuclear spin species I and $A_0 = \frac{\mu_0}{4\pi} \hbar \gamma_I \gamma_S$, with γ_I and γ_S the nuclear and electron gyromagnetic ratio, respectively.

Note that in Ref.[3] the assumption is made that $g_i(\omega+\Delta) \approx g_i(\omega)$, i.e., $\Delta \in (-\omega_{0I}, \omega_{0I})$ is small against the electronic inhomogeneous spectrum g_i. We do not make this approximation here and instead allow also for $g_i(\omega+\Delta) \not\approx g_i(\omega)$. In fact, for protons in the system under study ω_{0I} exceeds the width of the inhomogeneous EPR line in trityl[8] and for carbon nuclei there is barely an overlap ($g_i(\omega_{0I}) \gtrsim 0$).

We now take (36) from Wenckebach[3] that describes flow of electron dipolar energy, U_{NZ}, via TM, and we rewrite the exponentials therein using the hyperbolic tangent:

$$\frac{\partial U_{\mathrm{NZ}}}{\partial t} = -\frac{1}{2} N_S \omega_{0I} \int_{-\infty}^{\infty} \mathrm{d}\omega g_i(\omega) g_i(\omega - \Delta) \; W^{\mathrm{TM}} \tanh\left(\frac{1}{2} \omega_{0I} \left(\beta'_{\mathrm{NZ}} - \beta_I \right) \right) \tag{3}$$

with N_S the density of radical spins.

Now we insert the above expression (1) for W^{TM} into (3) and swap the order of integration. The integral without prefactors then is:

$$\mathcal{I}^{\mathrm{TM}} := \int_{-\omega_{0I}}^{\omega_{0I}} \mathrm{d}\Delta \int_{-\infty}^{\infty} \mathrm{d}\omega \frac{\left(\omega_{0I}^2 - \Delta^2\right)^{3/2}}{\omega_{0I}^3} g_D\left(\sqrt{\omega_{0I}^2 - \Delta^2}\right) g_i(\omega) g_i(\omega - \Delta), \tag{4}$$

This integral is calculated numerically with the assumptions for g_i and g_D given in the main manuscript.

Next we rewrite equation (3) and make use of the high-temperature approximation:

$$\begin{aligned}\frac{\partial U_{\mathrm{NZ}}}{\partial t} &= -\frac{1}{2} N_S \omega_{0I} \frac{\pi}{2}\ A^2\, \mathcal{I}^{\mathrm{TM}}\ \left(-\tanh\left(\frac{1}{2}\omega_{0I}(\beta'_{\mathrm{NZ}} - \beta_I)\right)\right)\\ &\approx \frac{1}{2} N_S \omega_{0I} \frac{\pi}{2}\ A^2\, \mathcal{I}^{\mathrm{TM}}\ \frac{1}{2}\omega_{0I}(\beta'_{\mathrm{NZ}} - \beta_I)\\ &= \frac{\pi}{8} N_S \omega_{0I}^2 (\beta'_{\mathrm{NZ}} - \beta_I)\ \frac{1}{10} A_0^2 N_I \frac{4\pi}{r_{\mathrm{ba}}^3}\ \mathcal{I}^{\mathrm{TM}}\\ &= N_I \omega_{0I}^2\ \frac{\pi^2}{20} A_0^2 \frac{N_S}{r_{\mathrm{ba}}^3} (\beta'_{\mathrm{NZ}} - \beta_I)\, \mathcal{I}^{\mathrm{TM}}\end{aligned} \tag{5}$$

Finally we take this change in energy per unit time of the non-Zeeman reservoir and set it equal to the sought-after TSF rate $\tau_{\mathrm{NZ}-I}$ with respect to the NZ heat capacity, i.e.,

$$\frac{\partial U_{\mathrm{NZ}}}{\partial t} = \frac{C_{\mathrm{NZ}}}{\tau_{\mathrm{NZ}-I}} (\beta'_{\mathrm{NZ}} - \beta_I)\,. \tag{6}$$

With $C_I = \frac{1}{4} N_I \omega_{0I}^2$ the heat capacity of the nuclear Zeeman reservoir, we find the triple spin flip rate:

$$\frac{1}{\tau_{\mathrm{NZ}-I}} = \frac{\pi^2}{5} A_0^2 \frac{N_S}{r_{\mathrm{ba}}^3}\ \frac{C_I}{C_{\mathrm{NZ}}}\ \mathcal{I}^{\mathrm{TM}} \tag{7}$$

VII. TWO-RESERVOIR RELAXATION

The nuclear reservoirs, with inverse temperatures β_{H} and β_{C}, and the Non-Zeeman (NZ) reservoir with β_{NZ}, all couple to one another and to the lattice. The heat exchange of the three reservoirs with each other and the lattice, depicted in Fig. 1 of the manuscript, is described by a set of three linear differential equations:

$$\frac{\partial \beta'_{\mathrm{H}}}{\partial t} = -\frac{1}{\tau_{\mathrm{NZ-H}}} \frac{C_{\mathrm{NZ}}}{C_{\mathrm{H}}} (\beta'_{\mathrm{H}} - \beta'_{\mathrm{NZ}}) - \frac{1}{\tau_{\mathrm{H-C}}} \frac{C_{\mathrm{C}}}{C_{\mathrm{H}}} (\beta'_{\mathrm{H}} - \beta'_{\mathrm{C}}) - \frac{\beta'_{\mathrm{H}}}{T_{1,\mathrm{H}}} \tag{8}$$

$$\frac{\partial \beta'_{\mathrm{C}}}{\partial t} = -\frac{1}{\tau_{\mathrm{NZ-C}}} \frac{C_{\mathrm{NZ}}}{C_{\mathrm{C}}} (\beta'_{\mathrm{C}} - \beta'_{\mathrm{NZ}}) + \frac{1}{\tau_{\mathrm{H-C}}} (\beta'_{\mathrm{H}} - \beta'_{\mathrm{C}}) - \frac{\beta'_{\mathrm{C}}}{T_{1,\mathrm{C}}} \tag{9}$$

$$\frac{\partial \beta'_{\mathrm{NZ}}}{\partial t} = +\frac{1}{\tau_{\mathrm{NZ-H}}} (\beta'_{\mathrm{H}} - \beta'_{\mathrm{NZ}}) + \frac{1}{\tau_{\mathrm{NZ-C}}} (\beta'_{\mathrm{C}} - \beta'_{\mathrm{NZ}}) - \frac{\beta'_{\mathrm{NZ}}}{T_{1,\mathrm{S}}} \tag{10}$$

Here $\beta'_i = \beta_i - \beta_{\mathrm{L}}$ for each reservoir ($i \in [\mathrm{H,C,NZ}]$) is the difference of the respective reservoir's inverse temperature $\beta'_i = 1/(k_B T_i)$ and the inverse lattice temperature, β_{L}.

The nuclear heat capacities, C_{H} and C_{C}, are calculated straightforwardly using Eq. (1) from the manuscript, and for the NZ reservoir C_{NZ} can be calculated using Eq. (2) once the electronic dipolar second moment is known.

The spin-lattice relaxation times for protons ($T_{1,\mathrm{H}}^{\square}$) are given by our measurements on neat pyruvic acid, where in absence of trityl all NZ related terms in the above equations drop out. The electronic spin-lattice relaxation time we estimate, based on EPR data[8], to be approximately constant in our field range, $T_{1,\mathrm{S}}^{-1} = 5\mathrm{s}^{-1}$.

The TSF rate couples the electron NZ reservoir to the proton ($\tau_{\mathrm{NZ-H}}^{-1}$) and carbon ($\tau_{\mathrm{NZ-C}}^{-1}$) reservoirs, and can be calculated as described in the previous section based on EPR spectral parameters.

The carbon reservoir is about $C_{\mathrm{H}}/C_{\mathrm{C}} = 64$ times smaller than the proton reservoir and relaxes much slower. So if we are only interested in the proton relaxation, we can limit our analysis, particularly at low fields, to protons and the NZ reservoir:

$$\frac{\delta \beta'_{\mathrm{H}}}{\delta t} = -\frac{1}{\tau_{\mathrm{NZ-H}}} \frac{C_{\mathrm{NZ}}}{C_{\mathrm{H}}} (\beta'_{\mathrm{H}} - \beta'_{\mathrm{NZ}}) - \frac{1}{T_{1,H}} \beta'_{\mathrm{H}} \tag{11}$$

$$\frac{\delta\beta'_{\mathrm{NZ}}}{\delta t} = +\frac{1}{\tau_{\mathrm{NZ-H}}}(\beta'_{\mathrm{H}} - \beta'_{\mathrm{NZ}}) - \frac{1}{T_{1,\mathrm{S}}}\beta'_{\mathrm{NZ}} \tag{12}$$

This can be rewritten in matrix form as in equation (3) of the main manuscript:

$$\frac{\delta}{\delta t}\begin{pmatrix}\beta'_{\mathrm{H}}\\ \beta'_{\mathrm{NZ}}\end{pmatrix} = \dot{\vec{\beta}} = \begin{pmatrix}-A & C\\ D & -B\end{pmatrix}\cdot\vec{\beta} \tag{13}$$

with the relaxation matrix entries $A = T_{1,\mathrm{H}}^{-1} + C_{\mathrm{NZ}}/C_{\mathrm{H}}\cdot\tau_{\mathrm{NZ-H}}^{-1}$, $B = \tau_{\mathrm{NZ-H}}^{-1} + T_{1,\mathrm{S}}^{-1}$, $C = \tau_{\mathrm{NZ-H}}^{-1}C_{\mathrm{NZ}}/C_{\mathrm{H}}$ and $D = \tau_{\mathrm{NZ-H}}^{-1}$.

Solving for the eigenvalues and corresponding eigenvectors we find relaxation rates:

$$R_{\mathrm{fast/slow}} = -\lambda_{\pm} = \frac{1}{2}\left(A + B \pm \sqrt{(A-B)^2 + 4CD}\right) \tag{14}$$

and with correspondings vectors:

$$\vec{v}_{\pm} = \begin{pmatrix}v_{\mathrm{H},\pm}\\ v_{\mathrm{NZ},\pm}\end{pmatrix} = \begin{pmatrix}-\frac{A-B\pm\sqrt{(A-B)^2+4CD}}{2D}\\ 1\end{pmatrix} \tag{15}$$

Any solution of (13) is then a linear combination of these two eigenmodes and we may rewrite:

$$\vec{\beta}(t) = a_{\mathrm{fast}}\cdot\vec{v}_{+}\cdot e^{-R_{\mathrm{fast}}t} + a_{\mathrm{slow}}\cdot\vec{v}_{-}\cdot e^{-R_{\mathrm{slow}}t} \tag{16}$$

In our experiments we expect the NZ reservoir to be initially at lattice temperature, i.e., $\beta'_{\mathrm{NZ}}(t=0) = 0$, such that from our initial conditions we find that $a_{\mathrm{fast}} = -a_{\mathrm{slow}}$, since $v_{\mathrm{NZ},+} = v_{\mathrm{NZ},-} = 1$. Combined with the initial proton inverse temperature $\beta'_{\mathrm{H}}(0)$, we can introduce normalized coefficients with respect to protons:

$$c_{\mathrm{H,fast/slow}} = \pm\frac{A - B \pm\sqrt{(A-B)^2+4CD}}{2\sqrt{(A-B)^2+4CD}} \quad , \quad c_{\mathrm{NZ,fast/slow}} = \mp\frac{2D}{2\sqrt{(A-B)^2+4CD}} \tag{17}$$

and rewrite the combined relaxation of the two reservoirs:

$$\vec{\beta}(t) = \begin{pmatrix}\beta'_{\mathrm{H}}(t)\\ \beta'_{\mathrm{NZ}}(t)\end{pmatrix} = \beta'_{\mathrm{H}}(0)\left(\begin{pmatrix}c_{\mathrm{H,fast}}\\ c_{\mathrm{NZ,fast}}\end{pmatrix}\cdot e^{-R_{\mathrm{fast}}t} + \begin{pmatrix}c_{\mathrm{H,slow}}\\ c_{\mathrm{NZ,slow}}\end{pmatrix}\cdot e^{-R_{\mathrm{slow}}t}\right) \tag{18}$$

In order to compare the biexponential relaxation with the experimentally determined single exponential rate we calculate the effective combined relaxation rate

$$R_{\mathrm{H,comb}}(t) = -\frac{\dot{\beta}_{\mathrm{H}}(t)}{\beta'_{\mathrm{H}}(t)} = \frac{R_{\mathrm{slow}}\cdot c_{\mathrm{NZ,slow}}\cdot e^{-R_{\mathrm{slow}}t} + R_{\mathrm{fast}}\cdot c_{\mathrm{NZ,fast}}\cdot e^{-R_{\mathrm{fast}}t}}{c_{\mathrm{NZ,slow}}\cdot e^{-R_{\mathrm{slow}}t} + c_{\mathrm{NZ,fast}}\cdot e^{-R_{\mathrm{fast}}t}} \tag{19}$$

and evaluate it at the time we measured it, i.e., at $t = T_{1,\mathrm{H}}^{\blacksquare}$.

For a validity check beyond comparing $R_{\mathrm{H,comb}}(t = T_{1,\mathrm{H}}^{\blacksquare})$ with the experimentally measured rates, we can now also have a look at what these derived relaxation rates give for limiting cases in the following subsections.

1. Slow relaxation in the high-field limit

For higher fields $C_{\mathrm{NZ}}/C_{\mathrm{H}}$ vanishes such that $C = 0$. We find $R_{\mathrm{fast/slow}} = \frac{1}{2}(A + B \pm |B - A|)$ and $c_{\mathrm{H,fast/slow}} = (B - A \mp |B - A|)/(2|B - A|)$. Here $|B - A| = B - A$ since $B > A$ at high fields. So the fast mode no longer contributes to the proton relaxation ($c_{\mathrm{H,fast}} = 0$) and protons relax slowly ($c_{\mathrm{H,slow}} = 1$) with $R_{\mathrm{slow}} = T_{1,\mathrm{H}}^{-1}$. This corresponds to the experimental observations that proton relaxation rates in neat and doped PA converge at higher fields.

2. Fast relaxation in the low-field limit

For lowest fields the NZ heat capacity exceeds that of the proton reservoir ($C_{\mathrm{NZ}}/C_{\mathrm{H}} \gg 1$), while the TSF rate is small $\tau^{-1}_{\mathrm{NZ-H}} \ll 1$, so that we approximate:

$$(A-B)^2 + 4CD = \left(T^{-1}_{1,\mathrm{H}} + C_{\mathrm{NZ}}/C_{\mathrm{H}} \cdot \tau^{-1}_{\mathrm{NZ-H}} - \tau^{-1}_{\mathrm{NZ-H}} - T^{-1}_{1,\mathrm{S}}\right)^2 + 4\tau^{-2}_{\mathrm{NZ-H}} C_{\mathrm{NZ}}/C_{\mathrm{H}}$$
$$\approx \left(T^{-1}_{1,\mathrm{H}} + C_{\mathrm{NZ}}/C_{\mathrm{H}} \cdot \tau^{-1}_{\mathrm{NZ-H}} + \tau^{-1}_{\mathrm{NZ-H}} - T^{-1}_{1,\mathrm{S}}\right)^2$$

and we can rewrite:

$$R_{\mathrm{fast/slow}} = \frac{1}{2}\left(A + B \pm \sqrt{(A-B)^2 + 4CD}\right)$$
$$\approx \frac{1}{2}\left(T^{-1}_{1,\mathrm{H}} + C_{\mathrm{NZ}}/C_{\mathrm{H}} \cdot \tau^{-1}_{\mathrm{NZ-H}} + \tau^{-1}_{\mathrm{NZ-H}} + T^{-1}_{1,\mathrm{S}} \pm |T^{-1}_{1,\mathrm{H}} + C_{\mathrm{NZ}}/C_{\mathrm{H}} \cdot \tau^{-1}_{\mathrm{NZ-H}} + \tau^{-1}_{\mathrm{NZ-H}} - T^{-1}_{1,\mathrm{S}}|\right) \tag{20}$$

Thus, we find rates:

$$R_{\mathrm{slow}} \approx T^{-1}_{1,\mathrm{S}}\ , \qquad R_{\mathrm{fast}} \approx T^{-1}_{1,\mathrm{H}} + C_{\mathrm{NZ}}/C_{\mathrm{H}} \cdot \tau^{-1}_{\mathrm{NZ-H}} + \tau^{-1}_{\mathrm{NZ-H}} \approx C_{\mathrm{NZ}}/C_{\mathrm{H}} \cdot \tau^{-1}_{\mathrm{NZ-H}} \tag{21}$$

With the same approximations, one finds the proton coefficients for two modes to be $c_{\mathrm{H,fast}} \approx 1$ and correspondingly $c_{\mathrm{H,slow}} \approx 0$ for lowest fields, so that the fast mode relaxation dominates at low fields.

Note that this limiting scenario for lowest fields corresponds to the slow thermal mixing described by (8.82) in Ref.[9].

3. Interpretation of the two-mode relaxation

Whenever two reservoirs that are in contact to an external bath, are also rapidly exchanging energy with each other, one will find a fast internal relaxation mode between the two and a slower mode with which the two-reservoir system as a whole equilibrates with the lattice. Note that the fast mode does not generally equilibrate the two reservoirs. Rather, it balances the two reservoirs' inverse temperatures to the point where both relax with the slow mode relaxation rate ($\dot{\beta}_{\mathrm{H}}(t)/\beta_{\mathrm{H}}(t) = \dot{\beta}_{\mathrm{NZ}}(t)/\beta_{\mathrm{NZ}}(t)$).

In the considered field-cycling experiments, the proton spins are allowed to reach thermal equilibrium at a magnetic field of 2 T and a temperature of 4.2 K. Subsequently the field is ramped down rapidly to a (much) smaller evolution field strength. At this field, initially the proton reservoir is much 'colder' than the lattice, whereas the electron NZ reservoir is at lattice temperature. The fast mode balances this two-reservoir-system internally by heating the protons at the expense of cooling the NZ reservoir. The slow mode describes the heating of both reservoirs to the lattice temperature.

At lower evolution fields, the electron NZ reservoir's heat capacity becomes more significant. The NZ reservoir can therefore exchange more heat with the proton spins in balancing temperatures in the fast mode, leading to a more pronounced effect observable in the proton relaxation.

At higher evolution fields, the electron NZ reservoir's heat capacity becomes negligible. The NZ reservoir therefore exchanges much less heat with the proton spins in the fast mode. So the observable effect on the proton relaxation of the fast mode is negligble, whereas the unobserved sudden cooling of the NZ reservoir should be significant.

VIII. OX063 EPR SPECTRUM AND TSF RATES

For some of our analysis we rely on electron paramagnetic resonance (EPR) data from the literature, in particular the electron $T_{1,\mathrm{S}}$ and the EPR spectrum. Both were reported for 15 mM trityl(OX063) by Lumata et al.[8]. In Fig. S4 (a-c) we compare their measured EPR X-band spectrum with three differently modelled spectral densities and display the resulting TSF rates for protons and carbon nuclei in Fig. S4 (d-f).

The modelled EPR spectra are convolutions of the inhomogeneous spectrum $g_i(\omega)$ and the (homogeneous) spin-spin interaction spectrum $g_D(\omega)$. For inhomogeneous spectrum $g_i(\omega)$ we use in all three cases a Gaussian with width corresponding to field-dependent linewidths displayed in Fig. 4(b) of Lumata et al.[8]. The models differ in their spin-spin interaction spectrum.

(i) Monte Carlo simulations[10] yield a (random) homogenous spatial distribution that gives a narrow $g_D(\omega)$ as described in the manuscript, which is shown in Fig. S4 (a). Consequently its second moment is quite small, corresponding to a frequency of only $\sqrt{M_2}/2\pi = 2.7\,\mathrm{MHz}$.

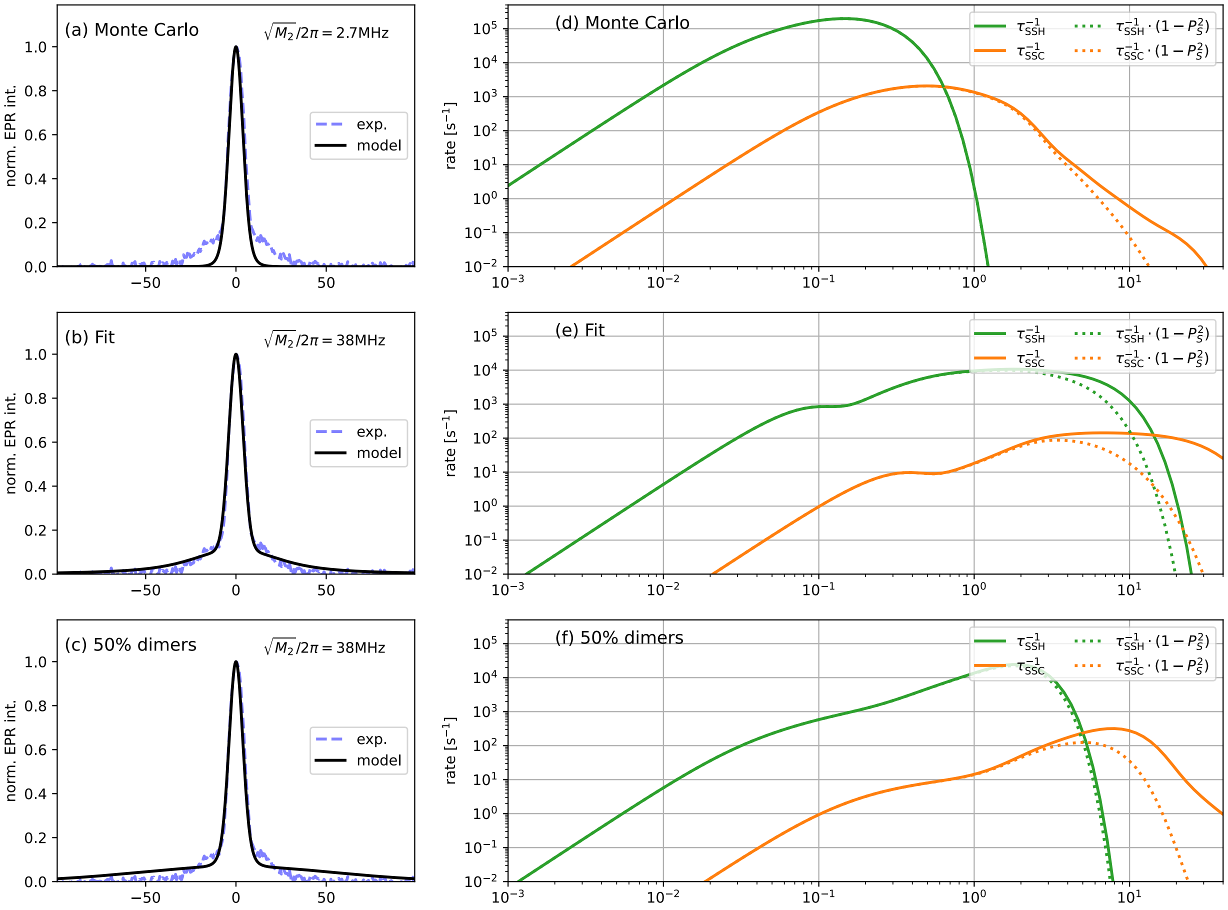

FIG. S4: (a-c) Trityl EPR spectrum (black solid lines) of three models (i-iii) discussed in the text at $B = 0.35\,\mathrm{T}$ compared to measured spectral density of 15 mM OX063 in 1:1 water/glycerol at 0.35 T and 100 K (blue dashed line), reproduced from Ref.[8] with permission. (d-f) Field-dependent TSF rates for heat exchange of the electron NZ reservoir with protons (green lines) and carbon nuclei (orange lines) for models (i-iii). At 4 K the substantial electron polarization $P_S = \tanh(\frac{\hbar}{2}\gamma_s B_0 \beta_s)$ above about 2 T suppresses TSFs (dotted lines) with a factor $(1 - P_S^2)$.[9] Nonetheless, by partial saturation ($P_S < 1$) of the EPR line with microwave irradiation a heat exchange via TSFs is still possible to higher fields (solid lines for $P_S = 0$).

Our analysis implies a much larger second moment ($\sqrt{M_2}/2\pi = 38\,\mathrm{MHz}$), which is attributed to the propensity of radicals to cluster.

Since various scenarios for $g_D(\omega)$ are possible, we give two examples. Both give the required second moment and, at least in the investigated field range, similar TSF rates that are consistent with our data.

(ii) The spin-spin spectrum we use in the main manuscript, displayed in Fig. S4 (b), is an attempt to fit the spectrum Lumata et al. reported at 350 mT, while also providing the required second moment. It is the sum of a narrow Gaussian ($\Delta_G/2\pi = 2\,\mathrm{MHz}$) and a broad Voigt profile ($\Delta_G/2\pi = 12\,\mathrm{MHz}$, $\Delta_L/2\pi = 21\,\mathrm{MHz}$) with an intensity (area) ratio $A_{\mathrm{Gauss}}/A_{\mathrm{Voigt}} = 0.92$. To limit the spread of the Voigt profile to reasonable values for dipolar interaction we multiplied it with a peak normalized Gaussian ($\Delta_G/2\pi = 200\,\mathrm{MHz}$).

(iii) Another possible spin-spectrum consistent with the experimental NMR data ($\sqrt{M_2}/2\pi = 38\,\mathrm{MHz}$) is a broad dipolar spectrum ($\Delta_G/2\pi = 54\,\mathrm{MHz}$) for half the trityl molecules (equivalent to 50% dimers), and a narrow spectrum for the homogeneously distributed radicals, described by Monte Carlo based parameters.

We briefly discuss contributions of natural abundance ^{13}C hyperfine couplings to the homogeneous spectrum. For the central carbon C_1, the hyperfine coupling tensor has been reported for the closely related Finland trityl as ($A_x = A_y = 18 \pm 2\,\mathrm{MHz}$, $A_z = 162 \pm 1\,\mathrm{MHz}$)[11]. The (averaged) solution state couplings for the next nearest C_2 carbon (three-fold degeneracy) is 32 MHz, and the coupling to $C_{3,3}$ (six-fold degenerate) is 25.4 MHz[11]. After scaling these

numbers with the respective degeneracy and the abundance of carbon (0.01), it is seen that the hyperfine couplings cannot provide the required second moment.

Note that the homogeneous spectrum could be simulated if the spatial distribution of radicals were known. However, the reverse is not true. A detailed field-dependent, pulsed EPR study would be required to elucidate this distribution.

IX. PYTHON CODE FOR NUMERICAL TSF CALCULATIONS

We use the standard Python libraries, and the spindata module, which may be installed from the Python Package Index PyPI.

```
import numpy as np
from scipy.integrate import dblquad
from scipy.constants import mu_0, N_A, hbar
import scipy as sp
import spindata
```

The TSF rate is calculated using the function:

```
def TSF(B, nuc, diffBarr, concentration, nucConc, gD, gI, rescale = True, which = "both",
        CNZ = 0, CCE = 0):
    """Calculate the triple spin flip-rate
    B: Field in Tesla
    nuc: Nucleus
    radDiameter: diameter of the radical in meter
    gD: function specifying the spin-spin spectrum,
        has to take an optional keyword argument field
    gI: function specifying the inhomogeneous spectrum,
        also has to take a keyword argument field.
    """
    preFactor = np.pi**2/5*(A(nuc))**2*molarToNumberDensity(concentration)/(diffBarr)**3
    integral = I(B, nuc, gD, gI, which=which)

    if CNZ == 0:
        CNZ = C_NZ("E", concentration, secMom(gD,3*100e6*2*np.pi,),"SI")

    #print('dipolar Freq is ',  (secMom(gD,3*100e6*2*np.pi))**0.5/(2*np.pi*1e6), 'MHz')

    scaleFactor = 1
    if rescale:
        heatCap = heatCapacity(nuc, nucConc, B,"SI")
        scaleFactor = heatCap/CNZ
    return scaleFactor*preFactor*integral
```

where the integral is calculated numerically, using scipy's `dblquad`:

```
def I(B, nuc, gD, gI, which = "both"):
    g = spindata.gamma(nuc)
    omega0I = g*B

    def integrand(omega, Delta, which=which):
        factorDict = { "both" : np.sqrt(omega0I**2 - Delta**2)/omega0I,
                       "TM" : (omega0I**2 - Delta**2)**(3./2)/(omega0I**3),
                      "CE" : Delta**2*(omega0I**2 - Delta**2)**(1./2)/(omega0I**3) }

        v = (gI(omega, field=B)
                  *gI(omega-Delta, field=B)
                  *factorDict[which]
                  *gD(np.sqrt(omega0I**2 - Delta**2)))
```

```
        return v

    # this is unstable though, and we have hence set the boundaries to 1000 MHz
    bound = 2*np.pi*1000e6
    # the outer integral is over Delta and goes from - to + omega0I,
    # and these bounds have to be given as a function
    res = dblquad(lambda Delta, omega: integrand(omega,Delta),  -bound, bound,
                  lambda k1: -omega0I, lambda k2: omega0I)

    return res[0]
```

For the inhomogeneous EPR spectrum $g_i(\omega)$ we use in all three cases (i-iii) a Gaussian. The width corresponds to data reported by Lumata et al.[8] (displayed in their Fig. 4(b)), which give the expectedly linear increase with field, but with a non-zero offset. We interpolate their data for the full width at half maximum, and convert it to a Gaussian width Δ_i using $\sqrt{8 \ln 2} \cdot \Delta_i / 2\pi = 5.0\,\text{MHz} + 8.4\,\text{MHz/T} \cdot B$. The function used for $g_i(\omega)$ used:

```
def scaledGaussianOffset(offset, gradient):
        # input is          standard dev. in Hz, i.e. FWHM/2.355
    def spectrum(omega, field = 0):
        sigma = (offset + gradient*field)*2*np.pi
        return 1 / np.sqrt(2*np.pi*sigma**2)*np.exp(-omega**2 / (2*sigma**2))
    return spectrum
```

The spin-spin interaction spectrum $g_D(\omega)$ in model (i) with the Monte Carlo based parameters[10] is a normalized product of a Gaussian with a Lorentzian, with linewidths for our system of $\Delta_\mathrm{G}/2\pi = 8.48\,\text{MHz}$ and $\Delta_\mathrm{L}/2\pi = 1.18\,\text{MHz}$. This is given by the following function (,when setting DDratio=0):

```
def spinSpinSpectrumRatio(d0, concentration, DDwidth, DDratio):
    """
    d0: radical diameter.
    concentration: electron concentration in Mol
    """
    D0 = mu_0/(4*np.pi)*hbar*(spindata.gamma("E"))**2

    deltaG = 1 / np.sqrt(5)*1/d0**3*D0
    # print("deltaG / 2 pi: {:.2e}".format(deltaG/(2*np.pi)))

    nS = molarToNumberDensity(concentration)
    deltaL = (np.sqrt(2*np.pi/5)*2*np.pi/3*nS*D0
              *(1+ np.sqrt(np.pi)*(np.sqrt(np.pi) - 1)*2*np.pi/3*d0**3*nS))
    print("deltaL / 2 pi: {:.2e}".format(deltaL/(2*np.pi)))

    def LORENTZIAN(DELTA):
        return deltaL/(np.pi*(DELTA**2 + deltaL**2))

    def GAUSSIAN(DELTA):
        return 1/np.sqrt(2*np.pi*deltaG**2)*np.exp(- DELTA**2 / (2*deltaG**2))

    def broadGAUSSIAN(DELTA):
        return 1/np.sqrt(2*np.pi*(2*np.pi*DDwidth)**2)*np.exp(- DELTA**2 / (2*(2*np.pi*DDwidth)**2))

    def PRODUCT(DELTA):
        """ This is not normalized... """
        return LORENTZIAN(DELTA)*GAUSSIAN(DELTA)

    boundary = 20*min(deltaL, deltaG)

    points = np.linspace(-boundary, boundary, num = 10000)

    dG = 2*boundary/len(points)
```

```
    # print("dG/2*pi: {:.2e}".format(dG/(2*np.pi)))
    g0 = 1.0 / (dG*np.sum(PRODUCT(points)))

    def PRODUCTNORMALIZED(DELTA):
        return g0*PRODUCT(DELTA)
    g1 = 1.0 / (dG*np.sum(PRODUCTNORMALIZED(points)))
    #.print("G1: ", g1)

    def DDspecComb(DELTA):
        return (1-DDratio)*PRODUCTNORMALIZED(DELTA)+DDratio*broadGAUSSIAN(DELTA)
    return DDspecComb
```

The same function is used for $g_D(\omega)$ in model (iii), but here half the electrons are described by a broad Gaussian with $\Delta_G = 54\,\text{MHz}$ and we set DDratio=0.5 and DDwidth=54e6.

In model (ii) the function used for $g_D(\omega)$ is:

```
def spinSpinSpectrumVoigt(sigmaTip, ampTip, sigmaBase, deltaBase, ampBase, sigmaLim):
    def VOIGT(DELTA):
        return ampBase*sp.special.voigt_profile(DELTA, sigmaBase,deltaBase)

    def GAUSSIAN(DELTA):
        return 1/np.sqrt(2*np.pi*sigmaLim**2)*np.exp(- DELTA**2 / (2*sigmaLim**2))

    def GAUSSIANtip(DELTA):
        return ampTip/np.sqrt(2*np.pi*sigmaTip**2)*np.exp(- DELTA**2 / (2*sigmaTip**2))

    def PRODUCT(DELTA):
        """ This is not normalized... """
        return (VOIGT(DELTA)+GAUSSIANtip(DELTA))*GAUSSIAN(DELTA)

    boundary = 10*sigmaLim

    points = np.linspace(-boundary, boundary, num = 10000)

    dG = 2*boundary/len(points)
    # print("dG/2*pi: {:.2e}".format(dG/(2*np.pi)))

    g0 = 1.0 / (dG*np.sum(PRODUCT(points)))
    # print("G0: ", g0)

    def PRODUCTNORMALIZED(DELTA):
        return g0*PRODUCT(DELTA)

    g1 = 1.0 / (dG*np.sum(PRODUCTNORMALIZED(points)))
    #.print("G1: ", g1)

    return PRODUCTNORMALIZED
```

The prefactor A, the conversion from molarity to number density and the heat capacities are calculated as follows:

```
def A(nuc):
    return mu_0/(4*np.pi)*hbar*spindata.gamma("E")*spindata.gamma(nuc)

def molarToNumberDensity(concentrationMolar):
    return concentrationMolar*N_A*1000

def heatCapacity(spin, N, B, units = "frequency"):
    """c.f. Goldmann, p. 22"""
    gamma = spindata.gamma(spin)
    I = spindata.spin(spin)
```

```
    if units == "SI":
        preFactor = hbar
    else:
        preFactor = 1
    heatCapacity = preFactor*B**2*gamma**2*N*I*(I+1) / 3
    return heatCapacity

def C_NZ(spin, N, secondMoment, units="frequency"):
    gamma = spindata.gamma(spin)
    I = spindata.spin(spin)
    H_L = np.sqrt(secondMoment*5/3)
    if units == "SI":
        preFactor = hbar
    else:
        preFactor = 1
    heatCapacity = preFactor*H_L**2*N*I*(I+1)/3
    return heatCapacity
```

* michael.jurkutat@kit.edu
† benno.meier@kit.edu
[1] A. J. Horsewill and Q. Xue, Phys. Chem. Chem. Phys. **4**, 5475 (2002).
[2] D. T. Peat, M. L. Hirsch, D. G. Gadian, A. J. Horsewill, J. R. Owers-Bradley, and J. G. Kempf, Physical Chemistry Chemical Physics **18**, 19173 (2016).
[3] W. T. Wenckebach, Applied Magnetic Resonance **52**, 731 (2021).
[4] W. Wenckebach, Nuclear Instruments and Methods in Physics Research Section A: Accelerators, Spectrometers, Detectors and Associated Equipment **356**, 1 (1995).
[5] W. Wenckebach, Journal of Magnetic Resonance **299**, 124 (2019).
[6] W. Wenckebach, Journal of Magnetic Resonance **299**, 151 (2019).
[7] W. Wenckebach, A. Capozzi, S. Patel, and J. Ardenkjær-Larsen, Journal of Magnetic Resonance **327**, 106982 (2021).
[8] L. Lumata, Z. Kovacs, A. D. Sherry, C. Malloy, S. Hill, J. van Tol, L. Yu, L. Song, and M. E. Merritt, Phys. Chem. Chem. Phys. **15**, 9800 (2013).
[9] W. Wenckebach, *Essentials of Dynamic Nuclear Polarization* (Spindrift Publications, 2016).
[10] W. T. Wenckebach and Y. Quan, Journal of Magnetic Resonance **326**, 106948 (2021).
[11] W. Moore, J. E. McPeak, M. Poncelet, B. Driesschaert, S. S. Eaton, and G. R. Eaton, Journal of Magnetic Resonance **318**, 106797 (2020).
