# Supplementary material for: Radical-Induced Low-Field 1H Relaxation in Solid Pyruvic Acid Doped with Trityl-OX063
Source: J Phys Chem Lett. 2022 Oct 31;13(44):10370–6. doi: 10.1021/acs.jpclett.2c02357 (PMC9661535; doi:10.1021/acs.jpclett.2c02357)
Supplement: Supplementary file 2 — jz2c02357_si_002.pdf [file jz2c02357_si_002.pdf]

jz-2022-02357w.R1

Name: Peer Review Information for "Radical-induced Low-field $^{1}H$ Relaxation in Solid Pyruvic Acid Doped with Trityl-OX063"

First Round of Reviewer Comments

Reviewer: 1

Comments to the Author

Dear authors

I find your work highly interesting and well worthy of publication in JPC Letters. However, I have some minor comments in the attached pdf that need attention before acceptance. My main concern is the weak support for the excessive dipolar broadening of the ESR line based on an assumption/model of aggregation. Further details are in the marked up attachments.

Best regards

Reviewer: 2

Comments to the Author

Jurkutat et al. show an analysis of 1H relaxation times in solid pyruvic acid doped with OX063 radicals at low temperature and at different magnetic fields. The paper describes both measured data as well as theory explaining observations. The paper overall is not very clearly written, it contains many redundant abbreviations (e.g., T1, 1H T1, T1,H^(dot), T1 (referred to 13C or electrons)) which are not always properly explained. Figure 2 is the same as Fig. 1. The presented paper may become a nice contribution to the field of hyperpolarized NMR, however, it does not warrant urgency necessary for publication in JPCL and better suited for a more specialized physical chemistry journal such that JPC (A,B,C). In case of publication in JPC, clear distinction between methods section, theory section, results section, discussion etc. would be necessary to improve the flow of the paper and make it more accessible for an interested reader.

Author's Response to Peer Review Comments:

Our responses (marked by '>>') to the Reviewers' comments

Reviewer 1 Comments:

"I find your work highly interesting and well worthy of publication in JPC Letters. However, I have some minor comments in the attached pdf that need attention before acceptance.

My main concern is the weak support for the excessive dipolar broadening of the ESR line based on an assumption/model of aggregation. Further details are in the marked up attachments."

>> We appreciate this concern and, as described below, we now discuss the dipolar broadening in greater detail in the revised manuscript.

.......................

Reviewer 1 Annotations to the Manuscript:

page 1, paragraph 2:

strike 'levels' in '... polarization levels in excess ...'

>> We agree and have rephrased accordingly.

page 1, paragraph 3:

‘narrow-band' not precise enough

>> We agree, and since we discuss this in detail later, we strike out “narrow-band” here.

page 2, Fig. 1 caption:

specify that neat PA temperature is the same

>> We agree and have rephrased accordingly.

page 2, paragraph 2:

"I would suggest using an open square as in the figure."

>> We thank the reviewer for the suggestion and have changed the notation to match symbols in the plots.

page 2, paragraph 6:

"Only two are mentioned. The electron Zeeman is missing, or correct to say "these two". Fig 2 does not show a sketch of the reservoirs. I do not think the sketch at the abstract illustrates the relevant reservoirs and parameters well either."

>> “These three” refers to the electron NZ and the two nuclear Zeeman reservoirs, which is evident now that we include the correct file for Fig. 2, see below.

page 2, paragraph 6:

"but isn't that the case when the field is lower than approx. 50 mT? I.e. for the low field points the electron Zeeman reservoir cannot be ignored?"

>> The possible differences in electron spin Zeeman energies are given by the inhomogeneous linewidth, which scales with the field like the nuclear Larmor frequency. The proton Larmor frequency exceeds the inhomogeneous EPR linewidth of trityl by a factor two, so the cross effect is negligible at any field. It is for this reason that trityl radicals are not suitable for proton hyperpolarization under D-DNP conditions.

page 2, Fig. 2:

same as Fig. 1

>> We now include the correct image file.

page 3, paragraph 1:

"It would be nice to explain this factor. It may not be clear to everyone that the concentration difference is a factor 4 (close to the gamma ratio by coincidence."

>> We agree and have made according changes.

page 3, last paragraph:

"There is some field dependence. How sensitive are the simulations to T1S?"

>> The strongest influence of a T1,S field dependence would be seen in the fast thermal mixing regime (dash-dot line in Fig. 3e and Eq. (7) ), where the electron T1,S is the bottle-neck. Here the data and calculations match very well. Conversely, the small deviations between model and data at low field cannot be attributed to a field-dependent T_1S, since here the model predicts slow thermal mixing (Eq. (8)).

page 5, paragraph 4:

"I find the explanation unsubstantiated. I accept that there could be clustering/ aggregation/ pairs. There is indication of that in several studies. But these pairs are only a small percentage (In Eq. 2, Ns would be a smaller number). I understand from the SM that the fraction is assumed substantial. However, the fit is not great (in my opinion). What is the effect of 13C hyperfine splittings in the ESR spectrum? The trityl is a large molecule and substantial 13C satellite intensities are present. Can this be modeled better? It could be interesting to investigate another trityl that may not cluster."

>> Clustering in trityl may be significant even at low concentrations. This is evident from substantial line-broadening observed in EPR at concentrations as low as 40 mM (J. Phys. Chem. Lett. 2020, 11, 3718−3723 ). Our analysis leads to an estimate of the second moment of the homogeneous line which is in good agreement with the experimental EPR spectrum (Fig. S4b). We now discuss the differences between models (ii) and (iii) in greater detail in the supplement.

It is true that the EPR spectrum could be simulated if the spatial distribution of radicals were known. However, the reverse is not true. A detailed field-dependent, pulsed EPR study would be required to elucidate this distribution.

On the other hand, 13C hyperfine splittings only give rise to a small contribution to the homogeneous line, as we now discuss in detail in the supplement.

We have also included these points in our rephrased argument for our model with increased homogeneous linewidth in the main manuscript.

……………………

Reviewer 1 Annotations to the Supplement:

page 5, equation (3) and following:

"why use the x symbol in this equation?" and "same here. Inconsistent use"

>> We agree and have now dropped the times symbol from the equations.

page 8, bottom:

"Define A: area or amplitude?"

>> The reference is to area, not amplitude, which we now specify explicitly.

------------------------

Reviewer 2 Comments:

"Jurkutat et al. show an analysis of 1H relaxation times in solid pyruvic acid doped with OX063 radicals at low temperature and at different magnetic fields. The paper describes both measured data as well as theory explaining observations. The paper overall is not very clearly written, it contains many redundant abbreviations (e.g., T1, 1H T1, T1,H^(dot), T1 (referred to 13C or electrons)) which are not always properly explained. Figure 2 is the same as Fig. 1. The presented paper may become a nice contribution to the field of hyperpolarized NMR, however, it does not warrant urgency necessary for publication in JPCL and better suited for a more specialized physical chemistry journal such that JPC (A,B,C). In case of publication in JPC, clear distinction between methods section, theory section, results section, discussion etc. would be necessary to improve the flow of the paper and make it more accessible for an interested reader."

>> We now use a more specific and consistent notation for the various T1 processes.

Furthermore, upon suggestion by Reviewer 1, when we refer to experimental data in neat and doped PA, we mark this with open and full squares, in exact correspondence with the symbols used for these data in the figures.
